# Neurological Sequelae of Acute Hydrogen Sulfide Poisoning: A Literature Review, Controversies, and Knowledge Gaps

**DOI:** 10.3390/neurolint17050071

**Published:** 2025-05-06

**Authors:** Wilson K. Rumbeiha, Dong-Suk Kim

**Affiliations:** Department of Molecular Biosciences, School of Veterinary Medicine, University of California, Davis, CA 95616, USA; dskkim@ucdavis.edu

**Keywords:** hydrogen sulfide, cyanide, acute exposure, neurological sequelae, delayed effects, brain lesions, anoxia, hypoxia, neuropsychological effects, vegetative state

## Abstract

Hydrogen sulfide (H_2_S) is a highly potent toxic gas, and the brain is a primary target organ following acute intoxications. Accidents and misuse of this gas for nefarious purposes, i.e., bioterrorism, are causes for concern regarding acute poisoning. The immediate effects of acute H_2_S poisoning are well known. Numerous publications have reported neurological sequelae, including insomnia, persistent headaches, ataxia, cognition deficits, hearing impairment, dysarthria, and neuropsychiatric behaviors, among survivors of acute H_2_S poisoning. However, this subject remains controversial. The goal of this study was to review the literature on acute H_2_S-poisoning-induced neurological sequelae and on animal models to determine prevalence and knowledge gaps. We also reviewed the literature on cyanide-induced neurological sequelae. The results of large population studies indicate that the majority of victims of acute H_2_S poisoning survive. There is a lack of patient follow-up and standardized neuropsychological, neurological, and neuroimaging for accurate assessments. We observed flaws in animal models that failed to recapitulate the severe neurotoxicity induced via the inhalation route. We observed a paucity of literature on cyanide-induced neurological sequelae. In contrast to cyanide-induced sequelae, predominantly characterized by Parkinsonian-like motor behavioral deficits, H_2_S patients exhibit mostly cognition deficits, speech impairment, and neuropsychological effects. This first comprehensive review of neurological sequelae induced by H_2_S and cyanide poisonings identified knowledge gaps in the prevalence of these sequelae and cellular and molecular mechanisms underlying them. It is unclear whether these sequelae are reversible. There are no FDA-approved drugs for the prevention or treatment of these sequelae. Notably, patients who received life-saving therapy still developed delayed neurological sequelae.

## 1. Introduction

Hydrogen sulfide (H_2_S) is a highly toxic gas used as a raw material in industry. It is transported and stored in bulk for this purpose [1]. H_2_S is classified as a blood agent because it inhibits cellular respiration and shares some similar toxic metabolic properties with cyanide and azide [2]. There are also multiple sources of H_2_S in the environment, and it is also a hazard in >80 occupational settings [1]. For example, it is a byproduct of industrial processes, e.g., in the natural gas and petroleum industry, and is produced naturally as a result of anaerobic decomposition of human and animal sewage. These are some of the many sources of accidental acute exposures to acute H_2_S poisoning. It is estimated that there are more than 1000 reports of acute exposures to H_2_S each year [3], and globally, the number of cases is likely to be much higher. H_2_S also has a history of use as a chemical weapon [4]. Components to make this lethal gas have been found in Islamic State camps [5] and were recently used in a foiled terrorist incident in Australia [6]. It is easily generated from commonly available materials [4,5,7]. As a result of the latter, H_2_S has been employed as a suicide agent in confined spaces, such as cars and apartments, endangering bystanders and/or first responders [8,9,10,11]. Because of its lethality and a history of use as a chemical weapon, there are concerns about the potential misuse of H_2_S as a weapon of mass destruction (bioterrorism), which could result in significant civilian casualties with high morbidity and mortality [5,7]. Currently, there are no drugs for reducing mortality and morbidity arising from acute H_2_S poisoning, including the treatment and prevention of neurological sequelae.

Although there is a large body of literature on the toxic effects of acute H_2_S poisoning, knowledge on the neurotoxic mechanisms of acute poisoning by this gas, both immediate and delayed, is still evolving. Commonly cited neurotoxic mechanisms include the inability of cells to utilize oxygen (histotoxic hypoxia) or hypoxia resulting from oxygen deficiency caused by poor gaseous exchange in the lungs, triggered by H_2_S-induced pulmonary edema, hypotension negatively impacting blood flow to the brain, and direct toxicity of the gas on brain cells. H_2_S is also a well-known inhibitor of cytochrome c oxidase (CCO), a terminal enzyme in the electron transport chain responsible for proton gradient to drive ATP generation [12,13]], resulting in energy deprivation and cell death. The brain is particularly vulnerable to this biochemical lesion because it has limited alternative pathways to generate the necessary energy [1]. Oxidative stress, neurotransmitter imbalances, calcium dysregulation, and altered neural network patterns are some of the other cited mechanisms of H_2_S-induced neurotoxicity [12,13,14,15,16,17,18,19,20,21]. The clinical phenotype of the immediate effects of acute H_2_S poisoning is well known and is characterized by a sudden loss of the sense of smell, irritation, sudden collapse (knockdown), coma, seizures, reduced breathing rate, dyspnea, and death within minutes following exposure to H_2_S at concentrations exceeding 500 ppm [12,13,22,23,24]. However, much less is known about the delayed neurological sequelae of acute H_2_S poisoning, including their prevalence, whether they are reversible or permanent, and the basic cellular and molecular mechanisms underlying the wide array of reported neurological sequelae, including learning and cognition deficits, postural instability predisposing to falls, insomnia, anxiety, depression, aggravated seizures, slurred speech, hearing impairment, and persistent headaches, among others [25,26,27,28,29,30,31,32,33,34,35,36,37,38,39,40]. Considering that neurological diseases are a major cause of morbidity and mortality worldwide, and that the number of these cases is increasing each year [41,42], it is important to understand the role of environmental factors in the pathogenesis of these diseases, including that of acute H_2_S poisoning. This knowledge is essential for developing therapeutic interventions to reduce the mortality and morbidity of acute H_2_S poisoning and potentially other toxicants, like cyanide and azide, which partly share similar neurotoxic mechanisms. The goal of this literature review was to gain a better understanding of the nature of neurological sequelae induced by acute H_2_S poisoning in humans, their prevalence, and the underlying mechanisms, if known. Since cyanide and H_2_S are both classified as blood agents and both are of concern as potential bioterrorism agents, we included cyanide-induced neurological sequelae in this review.

### Methodology

To the best of our knowledge, we are not aware of any comprehensive review of the literature on acute H_2_S or cyanide-induced neurological sequelae. Our review covered the peer-reviewed literature from 1950 to 2024, and we used the PubMed and Web of Science databases. For H_2_S, we used the following search strings for this review: “hydrogen sulfide” AND “sequelae”, “Hydrogen sulphide” AND “Sequelae”, “hydrogen sulfide poisoning” AND “brain injury”, “hydrogen sulphide poisoning” AND “brain injury”, “hydrogen sulfide” AND “neurological”, “hydrogen sulfide and sequelae” and “hydrogen sulphide and sequelae”. From 1 January 1950 to 31 December 2024, we found a total of 38 publications. Because the focus of this review was on neurological sequelae of acute H_2_S poisoning, we excluded any publications that did not include brain or neurological sequelae. For example, publications that reported on lung or heart sequelae alone without brain sequelae were excluded. In addition, because the focus of this review was on acute H_2_S exposures, such as those encountered via nefarious acts or industrial accidents, we excluded any publications on chronic low-level exposures or repeated/multiple exposures. As for the inclusion criteria, we only included peer-reviewed publications that reported on individual or groups of cases of acute H_2_S poisoning with neurological sequelae and publications that reported neurological sequelae due to industrial accidents resulting in mass civilian casualties. From a total of 38 peer-reviewed publications, we retained only 19 publications that met our inclusion criteria, which are summarized in Table 1.

Secondly, we also reviewed the existing literature on animal models used to study neurological sequelae of acute H_2_S poisoning during the same period. For this, we used the following search strings: “hydrogen sulfide poisoning” AND “brain” AND “mouse”, “hydrogen sulfide poisoning” AND “Brain” AND “rat”, “hydrogen sulfide poisoning” AND “brain” AND “rabbit”, “hydrogen sulfide poisoning” AND “brain” AND “dog”, “hydrogen sulfide poisoning” AND “brain” AND “sheep” and “hydrogen sulfide poisoning” AND “brain” AND “monkey”. A total of 115 publications were found. We then excluded any publications that did not involve animal models or did not report on brain lesions and/or behavior. For our inclusion criteria, we only included peer-reviewed publications of original studies that reported on long-term outcomes on the brain and/or included “neurological sequelae”, “neurodegeneration”, or “brain lesions”. This left only 12 publications that met the inclusion criteria for this review.

For the acute cyanide-induced neurological sequelae review, we used the following search strings: “sequelae of cyanide poisoning”; “cyanide poisoning” AND “neuropsychiatric”; “acute cyanide poisoning” AND “neurological”; “cyanide-induced sequelae”; “cyanide” AND “neurological sequelae”; “cyanide” AND “neurodegeneration”; “cyanide poisoning” AND “brain”; and “cyanide” AND “neuropsychological”. From 1 January 1950 to 31 December 2024, we found a total of 106 publications. Because the focus of this review was on the neurological sequelae of acute cyanide poisoning, we excluded any publications that reported chronic cyanide exposures. We also excluded those that did not include brain or neurological sequelae. For the inclusion criteria, we considered original case reports that included “acute cyanide poisoning”, “brain lesions”, and “neurological sequelae”. Out of a total of 106 peer-reviewed publications, we retained only 7 publications that met our inclusion criteria, which are summarized in Table 2.

## 2. Results of the Literature Review on the Neurological Sequelae of Acute H_2_S Poisoning in Humans

We found at least three published large population studies of mass civilian exposures to H_2_S. In the 1950 industrial accident at a gas treatment plant in Poza Rica, Mexico, 22 people (6%) were killed and 320 (94%) were hospitalized [39]. The exposure was for about 20 min. Of the 320 hospitalized victims, medical professionals closely monitored only 47 of them for an unspecified period of time and used undisclosed evaluation techniques. Of these 47 closely observed, 4 developed neurological sequelae, including slurred speech (dysarthria) [one patient], neuritis of the acoustic nerve (two patients), and one epileptic suffered a marked worsening of his condition [39]. The second large population study involved a review of 152 clinical cases of acute H_2_S poisoning in China, which was published by Wang et al. in 1989 [43]. The victims in their study were acutely exposed to H_2_S in different ways. Some were exposed to H_2_S produced by a fire in a sulfide production facility, others were victims of acute H_2_S exposure from putrefaction, while the rest were exposed to H_2_S via a leakage in the pipelines transporting the gas or other sources. Their study showed a 5.3% mortality rate, and 39 (41%) of the 95 patients who were followed-up for 1–10 year developed neuropsychiatric sequelae [43]. The reported neurological sequelae in their particular study included brain fog, impaired memory, difficulty focusing, decreased motivation, mental fatigue (collectively known as mental asthenia), hysteria, “mental illness”, and a decorticate posture. Unfortunately, as in the case of the Poza Rica, Mexico, accident, the techniques used for the evaluation of patients for neurological sequelae were not reported. The third large population study was the 2003 accidental sour gas well blow-out in China, which caused 243 (2.7%) deaths and 9000 hospital visits and hospitalizations [55]. The individuals who died were exposed for about 30 min. Unfortunately, no long-term follow-ups were performed to evaluate the surviving victims for neurological sequelae. All three large population studies showed that the overall mortality rate ranged from 2.7% to 6%, implying that the majority of victims of acute H_2_S poisoning survive the accidents. Notably, Wang’s review paper on a large population supports the observation that long-term follow-up and assessment using neuropsychiatric testing indicates that neurological sequelae are common and are largely behavioral in nature because 41% of the 95 patients followed up for 1–10 year developed neurological sequelae [43]. Considering the high survival rate of victims of this highly neurotoxic gas, it is imperative that we investigate the potential for survivors of accidents involving acute H_2_S exposure to develop neurological sequelae, study and identify underlying cellular and molecular mechanisms, and develop drugs for prevention and/or treatment of victims to reduce morbidity and mortality.

**Figure 1:**
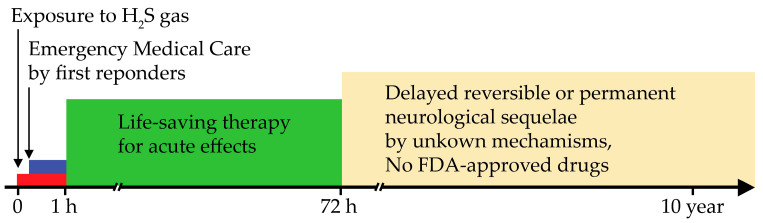
Schematic timeline of the development of delayed sequelae following acute exposure to hydrogen sulfide gas. Note that some survivors of acute poisoning progress to develop sequelae despite lifesaving therapy.

A summary of the other literature on neurological sequelae following acute H_2_S poisoning, most involving human case studies, is given in Table 1. The sex and age of the victims, if known, are given in the table. These delayed sequelae typically begin starting about 72 h postexposure and included movement disorders, such as walking with a spastic gait, slowed movement (bradykinesia), postural instability causing falls, learning and memory (cognition) deficits, deficits in executive planning functioning, hearing impairment, dysarthria, slowed mental and physical function (psychomotor retardation), sleep disorders, like insomnia, persistent headaches, neuropsychiatric disorders, aggravated pre-existing seizures, and, in the most severe cases, permanent vegetative states [26,27,28,29,30,31,32,33,34,35,36,40,43]. It was notable that not all individuals reported developing all these sequelae, as different individuals displayed some but not other symptoms. The severity of these neurological sequelae also varied from individual to individual for reasons that are not entirely known. Some victims became moribund and died weeks later, while others only displayed neuropsychological effects. Considering that the majority of the victims of acute H_2_S poisoning survive, and the majority of these survivors develop neurological sequelae, the health impact of these delayed sequelae can potentially be devastating by imposing a heavy burden on victims, caretakers, and the healthcare system, in general. Moreover, because this medical condition is understudied, currently, there are no FDA-approved drugs for the prevention or treatment of the neurological sequelae of acute H_2_S poisoning. Without knowledge of the underlying mechanisms, it is a challenge to develop target-specific therapeutic interventions. Moreover, some of the patients who received life-saving supportive treatment, including hyperbaric oxygen, still developed delayed neurological sequelae weeks or months later [46] (Figure 1).

**Figure 2:**
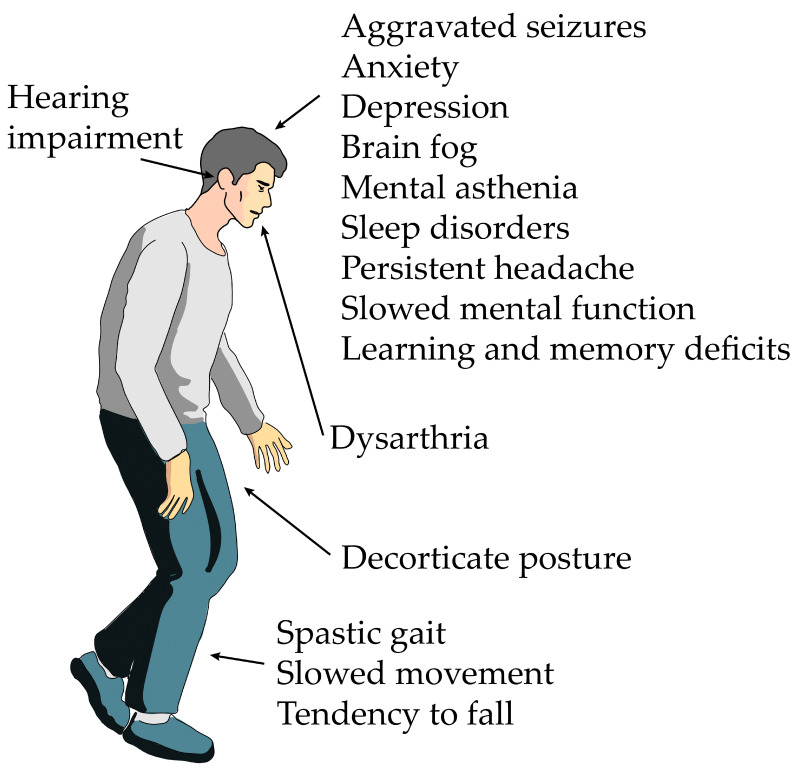
Schematic representation of the delayed effects of acute exposure to hydrogen sulfide gas. Patients display a wide range of symptoms. In severe cases, patients descend into permanent vegetative states. Not every patient develops all these symptoms. Different patients display different symptoms.

This literature review revealed several inconsistencies regarding how patients were evaluated for neurological sequelae following acute H_2_S poisoning. Notably, several reported human cases of acute H_2_S poisoning lacked adequate follow-up following the accidents to sufficiently evaluate the presence or absence of sequelae. Follow-up of patients for periods of 1–10 year would be ideal to conclusively determine whether neurological sequelae are present or not. Moreover, in some case reports, patient evaluations for neurological sequelae were not performed at all [55]. Another limitation was that when adequate follow-up was performed, the evaluation techniques and endpoints used to assess the presence of sequelae were variable and inconsistent. There was no standard patient evaluation approach. The endpoints assessed in various case reports included brain anatomical changes using imaging modalities, such as MRI, brain function changes using PET, neurological examinations, and neuropsychological examinations. Thorough evaluations would have involved the use of all these techniques for comprehensive patient evaluations, but this was not the case. Moreover, psychiatric examinations were suggested, but none of the reviewed case studies performed them. We also observed that the older literature [37,38] lacked the long-term follow-up of victims after hospital discharge and/or did not perform specialized neurological, neuropsychological, or brain imaging examinations ideal for determining long-term neurological impacts. These inconsistences may be some of the reasons why some authors have suggested that neurological sequelae after acute H_2_S poisoning are very rare [21]; however, others suggested that they are more common than reported because symptoms are easily overlooked or misinterpreted as functional psychiatric disorders or are undetected because of a lack of follow-up after hospital discharge [26,44]. For example, Haouzi et al. [21] cited Mooyaart et al. 2016 [37,56] to assert that sequelae are very rare. However, Mooyaart’s study focused on only eight victims who received cardiopulmonary resuscitation after the accidents and reported that six of these eight patients who received cardiopulmonary resuscitation (75%) did not have neurological sequelae. Yet, in the same Mooyaart study, they reported that 24 of the 54 victims (43%) survived, but whether the rest of the survivors were evaluated for neurological sequelae was not reported. Moreover, Mooyaart et al. only reviewed patient medical records and did not report the evaluation techniques used by physicians to assess the presence and types of neurological sequelae. They simply stated that no permanent damage was observed, which may be misleading [43]. Mooyaart’s study should be contrasted with other case reports in which appropriate long-term follow-up of victims and assessment techniques were performed, which showed that H_2_S-induced neurological sequelae are more common [26,34]. Specifically, Tvedt’s study on victims who were unconscious for 5–20 min and followed up for 5–10 year with neurological and neuropsychological examination showed that one patient had severe dementia and that memory and motor function were most affected [44]. They also reported that the two patients who were the most severely affected had pulmonary edema, which likely impacted neuropathology, emphasizing the importance of using the inhalation exposure route to recapitulate the lung–brain axis in the pathogenesis of H_2_S-induced neurological deficits. A schematic representation of some of the neurological sequelae of acute H_2_S poisoning is shown in Figure 2.

## 3. Distribution of Delayed Neurological, Anatomical, and Functional Lesions Following Acute H_2_S Poisoning

It was interesting that the lesions reported in the publications summarized in Table 1 were present only in select brain regions. Nam B et al. reported necrosis of the basal ganglia and motor cortex by MRI 30 days after an accident [27]. Matsuo F et al. showed bilateral cerebral and lentiform nucleus lesions in a patient in a chronic vegetative state who died 5 weeks after exposure [35]. Schneider JS et al. showed abnormal metabolism in the thalamus, basal ganglia, and temporal and inferior parietal lobes using PET 3 year after an accident [36]. The same study reported decreased metabolism in the putamen, amygdala, and hippocampus by SPECT 3.5 year after an accident. Tvedt B et al. (1991) showed cerebral atrophy and widening of the third ventricle using MRI and CT scans 5 year after an accident [26]. In 1987, Gaitonde et al. reported symmetric low densities of the basal ganglia by CT scan [57]. Thus, the cortex, thalamus, and basal ganglia are the brain regions that frequently develop anatomic lesions. Notably, however, functional changes involved additional brain regions, including the hippocampus, suggesting H_2_S causes functional impairment without necessarily inducing brain lesions [36]. This is significant because the absence of anatomical lesions does not imply the absence of disease. In other words, it is possible for victims of acute H_2_S poisoning to develop neurological and/or neuropsychiatric disease without necessarily developing brain lesions. Warenycia MW et al. [58] (1989) suggested that the inhibition of the monoamine oxidase enzyme is a sequela of acute H_2_S poisoning, which is responsible for increased brain catecholamines and serotonin levels. This could be one of the mechanisms of H_2_S-induced neuropsychological sequelae, but further evaluation is needed. It is also possible that the reported brain anatomical changes could drive the development of the various neurological sequelae reported in various survivors of acute H_2_S poisoning, as summarized in Table 1.

Other toxicants, such as cyanide and azide, were reported to share a common toxic mechanism with H_2_S poisoning by inhibiting CCO activity, blocking ATP, and causing energy deficit in the brain [59,60]. Also, brain ischemia and hypoglycemia share some mechanisms with H_2_S poisoning as they cause hypoxia and/or energy deficit [61]. Indeed, hypoxia is often cited as the cause of the brain lesions following acute H_2_S poisoning [27,62]. We observed the distribution of brain lesions following acute cyanide or azide poisoning, which is similar to that of acute H_2_S poisoning. However, it is perplexing that the resulting clinical phenotypes are not identical, with cyanide inducing predominantly Parkinsonian sequelae and H_2_S inducing more cognition and neurobehavioral deficits, followed by motor deficits.

## 4. A Review of the Literature on Animal Models

Different types of animal models have been used to study the neurotoxic effects of H_2_S. Unfortunately, only a few studies using animal models met the criteria for inclusion in this review, specifically original research on neurological sequelae arising from acute exposure to H_2_S. Of the 12 that met the inclusion criteria, the following 4 studies stand out [21,63,64,65]. Lund et al. conducted an inhalation H_2_S exposure study in Rhesus monkeys [63]. Some monkeys lived up to 10 days postexposure, exhibiting somnolence and impaired movement. Brain histology revealed necrosis in the cortex, basal ganglia, and brainstem and the loss of Purkinje cells in the cerebellum. They concluded that the Rhesus monkey recapitulates the human effects of acute H_2_S poisoning. Baldelli et al. used rats to investigate brain necrosis [64]. They injected sodium hydrosulfide (NaHS) intraperitoneally (IP) and euthanized all surviving rats on day 7 post-injection. Only one of three surviving rats developed necrosis in the cortex. They concluded that it is extremely difficult to induce brain lesions in rats with H_2_S. Sonobe et al. (2015) and Haouzi et al. (2020) also used rats to investigate the long-term effects of acute H_2_S poisoning and evaluated the effects of methylene blue (MB) to counteract these effects [21,65]. As for Baldelli, they injected NaHS IP and also sacrificed the rats on day 7 post-injection. The majority of the rats not treated with MB died. But 9% of the surviving non-treated rats were euthanized, and brain lesions were found in the cortex, thalamus, and basal ganglia. According to Sonobe, these lesions did not match the pattern of post-ischemic lesions. The other surviving rats performed poorly in the Morris water maze (MWM) test. MB showed some efficacy in reducing lesion severity and improved performance in the MWM test. They acknowledged a limitation of their model, i.e., IP injection vs. via inhalation exposure, as no lung lesions were observed in their model. Hence, this approach does not recapitulate the natural inhalation route of exposure.

In our lab, we use a whole-body inhalational mouse model that recapitulates the natural route of acute H_2_S poisoning in humans, which is a single inhalation exposure [12,14]. The observation period was up to 7 days post-inhalation exposure. Using this mouse model, we found lesions in the cortex, thalamus, basal ganglia, inferior colliculi, and brainstem [12,13,14,15,16,17,18,19,66]. Like other animal studies to date, we have not conducted long-term studies following up mice for 6 months or longer. Nonetheless, we successfully induced brain lesions with this model.

Overall, we made two major observations from the literature review involving the use of animal models, both involving flaws in the research approach. A major flaw we observed in the research approach is that most investigators use intraperitoneal (IP) or intravenous injections of H_2_S chemical donors [64,65,67,68,69,70,71,72,73,74]. Animal models using IP/IV routes for H_2_S exposure [64,65] have difficulty replicating the neurological lesions reported in numerous human case reports, as listed in Table 1 [26,27,28,29,30,31,32,33,34,35,36,37,38,39,43,44], or replicating the data generated using inhalation mouse models [12,13,14,15,16,17,18,19,66] or the Rhesus monkey inhalation study [63]. Therefore, research from animal models using IP/IV injections of NaHS and other H_2_S chemical donors should be interpreted with caution. Secondly, we observed that there are no animal studies on the natural history of acute H_2_S poisoning in the literature, specifically those mimicking long-term follow-up following acute H_2_S exposure in humans. All animal studies so far have lasted 7 days, except for the Lund monkey study, which lasted 10 days. Long-term studies using appropriate animal models are needed to study the natural history of a single acute H_2_S exposure, along with in-depth behavioral assessments. Such studies will generate novel knowledge on the sequelae of acute H_2_S poisoning in humans, identify the mechanisms involved, and provide the basis for drug discovery to prevent or treat both the anatomical and behavioral changes induced by this potent gas.

## 5. Results of the Literature Review on the Neurological Sequelae of Acute Cyanide Poisoning in Humans

Here, we summarize the results of our literature review on cyanide. Grandas et al. described a case of a 39-year-old man who attempted suicide by ingesting an unknown amount of sodium cyanide in April 1983 [47]. On hospital admission, he was unconscious and apneic. With assisted ventilation, he regained consciousness after an unspecified number of days. On admission, his CT scan was normal. However, a month after admission, a follow-up CT scan revealed lesions in the basal ganglia and frontal cortex. At that time, the patient manifested signs of Parkinsonism, including bradykinesia, resting tremors, and postural instability. The signs progressively worsened, and 4 year later, in 1987, CT scans showed atrophy of the cortex and bilateral lesions in the basal ganglia. He did not respond to standard therapy for Parkinsonism. His neurological sequelae were permanent.

In 1991, Messing reported a case of a 29 year old man who ingested potassium cyanide [48]. On admission, he was not breathing and was comatose. He developed dysarthria and other signs of Parkinsonism. CT scans and MRI imaging revealed lesions in the basal ganglia 5 months after attempting suicide. In 1993, Kasamo et al. reported neurological sequelae characterized by lesions restricted to the caudate nuclei and putamina using MRI in a patient who ingested potassium cyanide [49]. He was admitted comatose without spontaneous breathing and was treated with antidotes within 1 h of ingesting cyanide. These lesions were still present 9 months after he attempted suicide, but they regressed, and the patient recovered. Rosenow et al. reported two cases of attempted suicide in 1995 [50]. Case 1 was a 22 year old man, and Case 2 was a 43 year old man. Case 1 needed respiratory support for 4 hrs, while Case 2 was supported for 24 h. The 22 yr old manifested Parkinsonian syndrome 5 weeks after ingesting cyanide. He had a reduced motor reaction and reduced speed of information processing. MRI 9 weeks after exposure revealed lesions in the basal ganglia, cortex, cerebellum, and enlarged cerebral ventricles. Functional images with FDG-PET revealed hypometabolism in the same regions. Case 2 also manifested Parkinsonism, including ataxia, falls, and dystonia [50]. Almost 5 year after the incident, bilateral lesions were still present but restricted to the basal ganglia. Standard therapy with dopaminergic drugs did not cause significant improvement.

A case of neurological sequelae following the ingestion of choke cherries was reported by Pentore et al. in 1996 [51]. This involved a 56 year old woman who, upon admission to the hospital, had breathing difficulty and went into a coma. For 2 weeks, she was confused, disoriented, and agitated. About 30 days post-admission, she developed signs of Parkinsonism and reduced bilateral visual acuity. However, at 15 months post-admission, the patient had fully recovered [51]. In 2002, Rachinger et al. reported a case of a 35 year old female who presented comatose 10 min after ingesting cyanide [52]. She was on respiratory support. Twenty-one days after the incident, imaging studies revealed pseudolaminar necrosis along the sensorimotor cortex and discrete parts of the basal ganglia. These lesions were still present 6 weeks after the patient ingested cyanide, with hemorrhage in the basal ganglia.

Mohan et al. reported neuropsychological effects in a 22 year old woman who ingested cyanide [53]. The patient was followed over a 6 mo period. She self-presented at the hospital and was admitted and treated in the intensive care unit (ICU) for cyanide poisoning. She was on ventilation for 48 hrs. Around day 5 post-ingestion, she had deficits in episodic memory. This patient did not manifest any Parkinsonism signs, and a neurologic examination was normal at this early stage. Evaluation by a psychiatry team revealed impaired episodic memory and retrograde amnesia. She also had attention deficits. MRI neuroimaging revealed lesions in the basal ganglia, thalamus, and cerebral hemispheres and edema in the hippocampus. At 5 months, these lesions were still present, albeit smaller, but the hippocampus was reported to have atrophied. A neuropsychological assessment at 3 weeks post-injury revealed reduced cognitive functioning, such as recalling people’s names and problem-solving skills. She was reported to have a markedly impaired ability to assimilate and retain new information, as well as a severely impaired visual recall of geometric figures. However, these defects were significantly reversed at 5 months. Alqahtani et al. reported neuropsychiatric sequelae in a 45 year old woman who inhaled cyanide from a structural fire and developed cognitive deficits, dysarthria, dystonia, and altered sleep patterns 3 months after the incident [54]. They emphasized the need to conduct full neurological and intellectual evaluations to find neuropsychiatric sequelae in cases of acute cyanide poisoning because MRI imaging is not always diagnostic. In this case, the CT scan and MRI were normal.

An overall observation is that in human cases of H_2_S poisoning, exposure was by inhalation, but in cases of human cyanide poisoning, most reports were almost exclusively by oral ingestion. This may account for some of the differences noted between clinical signs reported as sequelae for cyanide and H_2_S.

## 6. Potential Mechanisms Underlying H_2_S-Induced Neurological Sequelae

There is also a major debate whether the neurotoxicity of H_2_S is a result of the direct effects of this gas on the brain or whether it is a result of secondary events, e.g., due to hypoxia [62]. However, there is sufficient evidence in the literature to show that acute H_2_S exposure induces hypoxia and that this is one of the mechanisms contributing to brain lesions [1,64,75]. However, questions on the downstream cellular and molecular mechanisms by which hypoxia triggers neuronal cell death and why neuronal cell death occurs only in selective brain regions rather than globally remain uninvestigated and unknown. Understanding these pathways is key to developing therapeutic interventions targeting critical pathways of cell injury and death. It is notable, however, that work from our laboratory and works of others affirm that H_2_S has direct effects on brain cells [16,20,62], causing direct brain injury.

There is a knowledge gap on the mechanisms leading to several neurological sequelae, such as dysarthria or hearing impairment. However, multiple pathways through which H_2_S causes damage to the brain, such as neuroinflammation, oxidative stress, cytochrome c oxidase inhibition, cell signaling disruption, calcium homeostasis dysregulation, neurochemical dysregulation, and others yet to be identified, may play a role in the pathogenesis of movement disorders, memory impairment, and neuropsychiatric disorders. However, the links have not been made. It is also noteworthy that there is a wide range of neurological sequelae in the human victims of acute H_2_S poisoning. Intriguingly, not all neurological sequelae are induced in all victims. There is no explanation for this observation in the reviewed literature. For example, some victims may experience dysarthria, while others may experience hearing impairment. Yet, others may develop neuropsychological effects and/or memory impairment. It is possible that individual factors, such as genetic predispositions, age, and sex, may explain why different individuals respond differently. It is noteworthy that this is not unique to H_2_S poisoning, as similar observations have been reported in victims of acute cyanide poisoning, but no explanations have been given [47,48,49,50,51,52,53,54,76,77,78,79,80,81]. Certainly, this should be one of the interesting areas of investigation. As reported by Fenga C. (2002), reduced cognition, depression, and personality changes can exist even when a neurological examination and neuroimaging are unremarkable, suggesting that the presence of anatomic brain lesions is not essential for the development of neurological sequelae [33]. Treating the neurological sequelae of acute H_2_S poisoning effectively needs research to specifically investigate the efficacy of potential drug candidates, such as methylene blue, in reducing some of these symptoms. As of now, this research is lacking.

Using an inhalation mouse model of acute H_2_S poisoning, our lab demonstrated altered dopamine, epinephrine, and glutamate levels [12,13,14]. Dysregulation of these and other neurotransmitters may contribute to the development of these sequelae. Acute H_2_S exposure caused increased dopamine and epinephrine concentration in several brain regions while, at the same time, gradually reducing glutamate levels. It is possible that such changes in neurotransmitter levels are responsible for some of the behavioral sequelae reported in humans. Dopamine is involved in coordinating movement, attention, and executive function. Epinephrine is involved in arousal and emotional memory. Glutamate is essential for long-term potentiation, which is essential for learning and memory. Moreover, H_2_S also causes acute lung injury (ALI) simultaneously [12,16]. The impact of pulmonary disorders on neurological health, commonly called the lung–brain axis, is well known [82,83]. Recent publications suggest that the mechanisms by which ALI contributes to brain injury are more complex than via hypoxia alone, which is commonly cited as a contributing factor [82,83]. Therefore, there is a critical knowledge gap in our understanding of the underlying molecular and cellular mechanisms, the minimum single toxic doses required to trigger them, the factors predisposing to neurological sequelae, whether the reported sequelae are reversible or not, and how to prevent or treat them.

**Figure 3:**
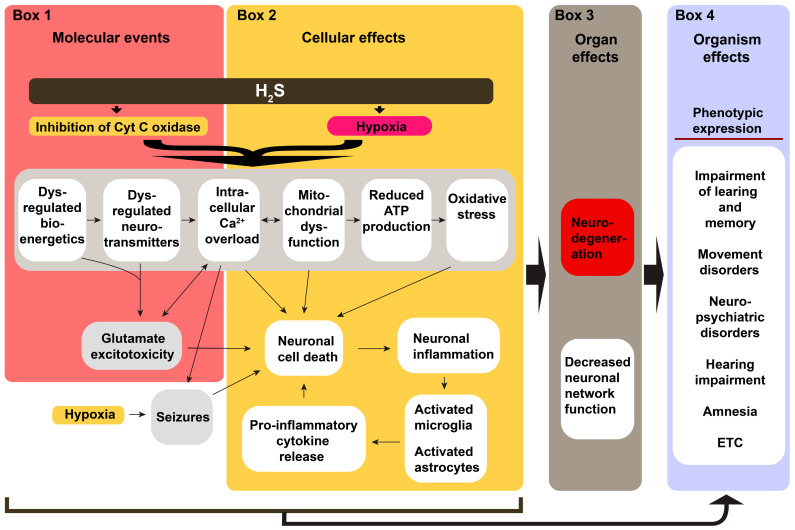
A summary of the overall hypothesis of H_2_S-induced neurotoxicity. Acute H2S poisoning causes acute death. However, some survivors of acute exposure develop long-term neurological sequelae (Box 4), including impaired memory and learning, movement disorders, such as ataxia, amnesia, hearing impairment, and neuropsychiatric disorders. Acute death is caused by impaired cytochrome c oxidase activity within the mitochondria, which causes impaired ATP synthesis and dysregulated energy (Box 1). Simultaneously, H2S interferes with neurotransmitters, such as dopamine, epinephrine, serotonin, and glutamate (Box 1). Altered energy balance also contributes to neurotransmitter imbalance (Box 1). H2S also causes hypoxia (Box 2). Hypoxia, in concert with the direct effects of H2S on cellular organelles, triggers calcium dysregulation. Collectively, these events trigger seizures, oxidative stress, and neuroinflammation (Box 2). These toxic cellular events cause neurodegeneration in select brain regions, such as the basal ganglia, thalamus, cortex, brainstem, and inferior colliculi. Neuronal cell death causes a disrupted brain network function. The neurologic sequelae can be caused by the events in Box 1, Box 2, Box 3, or a combination of these effects (see arrows at the bottom of the figure and from Box 3 to Box 4). Little research has focused on Box 4, and hence, currently, there are no FDA-approved drugs to prevent or treat these sequelae.

Our overall hypothesis on the mechanisms by which acute H_2_S exposure induces neurological sequelae is summarized in Figure 3. We hypothesize that the neurological sequelae of acute H_2_S poisoning are triggered by a cascade of multiple cellular mechanisms triggered by the direct effects of H_2_S on brain cells, inducing mitochondrial injury (energy failure), oxidative stress, calcium dysregulation, neurotransmitter imbalances, and neuroinflammation (Figure 3) [12,16,20,84]. Seizures cause secondary effects, including neurodegeneration [85]. Our secondary hypothesis is that acute H_2_S poisoning induces neurological sequelae via the lung–brain axis. In this regard, H_2_S-induced ALI contributes to the development of neurological sequelae via multiple mechanisms, including hypoxia (via pulmonary edema), pro-inflammatory cytokines, extracellular vesicles of lung origin, and the lung microbiome [82,83]. The published literature indicates that >80% of the survivors of acute respiratory distress develop neurological sequelae, including cognitive and emotional impairments, depression, anxiety, and persistent psychological distress [86,87,88,89,90,91,92]. We postulate that the combination of these direct and indirect mechanisms has additive or synergistic effects that cause the reported sequelae, particularly the neuropsychological effects. Thus, there is a huge knowledge gap and research opportunity on testing these hypotheses to generate knowledge on the underlying cellular mechanisms causing the neurological sequelae of acute H_2_S poisoning. This knowledge is essential for treating victims of acute H_2_S poisoning to reduce morbidity. Currently, there are no FDA-approved drugs for treating acute H_2_S poisoning. Some of the potential drugs currently being investigated that may have potential benefit to prevent or treat H_2_S-induced neurological sequelae are summarized in Table 3, but these deserve investigation.

## 7. Discussion

The work discussed in this review has broader implications. Both H_2_S and cyanide are environmental chemicals, and the environment has a role to play in the pathogenesis of neurological diseases. Cases of non-communicable neurological diseases are increasing annually as the population of older Americans increases [41,42,98,99]. Already, neurological diseases are reducing the quality of life of many Americans, accounting for significant mortality and morbidity, and contributing to escalating healthcare costs. Air pollution is also believed to contribute to mental disorders [100]. It is therefore essential that all environmental factors with the potential to contribute to the development of neurological and mental conditions, including dementia, cognition impairment, neuropsychological conditions, movement disabilities, etc., such as H_2_S and cyanide, are investigated and that the mechanisms are unraveled. This will allow for interventions, including the development of therapies to prevent and treat these neurological conditions and lower the disease burden.

Whereas the immediate short-term effects of acute H_2_S are well known, the literature on the prevalence of the neurological sequelae of acute H_2_S poisoning is meager and mixed. Some publications suggested that neurological sequelae are more common and likely underreported, while others suggested that they are very rare [21,26,44]. Moreover, Kilburn asserted that brief exposures to H_2_S are neurotoxic, and the effects are persistent [34]. We therefore conducted this review to understand the state-of-the-art knowledge on this subject. This is particularly important because both H_2_S and cyanide have been classified as potential chemical weapons or chemicals likely to be used for nefarious purposes. In this review, we observed that the neurological sequelae of acute H_2_S poisoning are common and are likely underreported in the literature. A wide array of neurological sequelae were reported in humans, including cognition impairment, locomotion challenges, such as ataxia, hearing impairment, sleep disturbances, emotional changes, seizures, and others. It was notable that different individuals developed different sequelae, but behavioral effects were more common among victims. We also observed that behavioral effects were present in the absence of anatomical brain changes, suggesting that brain lesions were not absolutely essential for the development of neurological sequelae.

We also noticed inconsistencies in the assessment and reporting of neurological sequelae in the published case reports. Most studies reported the immediate effects of acute poisoning but did not perform long-term follow-up studies, which are essential to uncover delayed sequelae. Another key deficiency we observed was the inconsistencies in patient evaluation for sequelae. Considering that neurological sequelae are many and varied, this suggests that different specialized medical expertise is required to accurately identify these sequelae. In the reviewed literature, when neurological sequelae were evaluated, the evaluations included brain anatomical or functional imaging, neurological examinations, or neuropsychological assessments in isolation. Notably, however, only a few studies performed patient evaluations using a combination of these techniques. The use of complementary techniques in patient evaluation would ensure comprehensive patient evaluations. We also observed that in some case reports, the patient assessment techniques used were not reported at all. This reduced the quality and value of such case reports. Moreover, some of the studies that failed to report the assessment techniques only involved a review of patient medical records. Overall, our observation is that as of now, there is a dearth of studies in which a long-term comprehensive assessment of victims of acute H_2_S poisoning was performed involving highly specialized medical specialists with expertise in radiology, neurology, and neuropsychology. As such, it is possible that the prevalence of neurological sequelae following acute H_2_S poisoning is currently underestimated. A similar problem was observed in cases of survivors of acute cyanide poisoning.

Regarding the literature using animal models, we observed a paucity of long-term studies investigating the neurological sequelae of a single acute H_2_S exposure. There are no studies on the natural history of single acute inhalation studies of H_2_S in animal models. Neurological sequelae take time to develop, and follow-up studies for 3 months to 2 years are needed, first, to identify the sequelae and, second, to determine whether or not these effects are reversible. Secondly, we observed a general trend in which animal studies used a flawed approach, i.e., injecting chemical donors of H_2_S IP or IV to study the effects of H_2_S on the brain and other tissues. Some of these studies reported difficulties in recapitulating brain lesions and other sequelae reported in humans. Such studies should be interpreted with caution and should be challenged. These studies do not recapitulate the natural route of human exposure, i.e., via inhalation, and do not cause ALI. Shortcuts using IP/IV injections of chemical donors negate the contributions of absorption through the olfactory route (nose–brain axis) and the contributions of acute respiratory distress induced by H_2_S (lung–brain axis) to the development of neurological sequelae. The potential contributions of the lung–brain axis via ALI to the pathogenesis of mood and other neuropsychological disorders reported in victims of acute H_2_S via inhalation in freely walking animal models cannot be ignored. It is our observation that more work needs to be performed using appropriate models and for suitable durations using multiple evaluation endpoints to fully determine the extent of neurological sequelae, the underlying mechanisms, and whether the sequelae are reversible or not. A short-term inhalation mouse model of a single acute H_2_S exposure has recapitulated locomotor impairment, neurochemical changes, and MRI lesions in select brain lesions of mice [12,14,15].

The strength of this study is that it is the first comprehensive review to evaluate publications on H_2_S-induced neurological sequelae and also to compare them with the sequelae of acute cyanide poisoning. Therefore, this work is novel. The limitation of this study is that the sample size (number of publications) of original publications in this field is small. This is also true for the literature on sequelae induced by acute cyanide poisoning. In case of the latter, it has been pointed out that acute cyanide poisoning is highly lethal, with only a few surviving victims, hence providing a limited opportunity to study sequelae.

## 8. Conclusions

H_2_S is a highly toxic gas that can cause mass mortality, as exemplified by industrial accidents in Mexico and China. Evidence from these published population studies suggests that the majority of victims in these acute H_2_S poisoning accidents survived and were hospitalized. There is also ample evidence from the literature, notably case studies of affected individuals, that survivors of acute H_2_S poisoning develop a variety of neurological sequelae weeks or months after acute exposure. These neurological sequelae are often, but not always, associated with anatomical changes in the brain, suggesting some are associated with neurotransmitter imbalances, network connectivity and signal transduction alterations, or other yet to be discovered mechanisms. A key concern is that currently, there are no FDA-approved drugs for the treatment of mass civilian casualties of acute H_2_S poisoning to prevent or cure neurological sequelae. This is because there is a knowledge gap on the types and prevalence of neurological sequelae, the cellular and molecular mechanisms involved, and whether these sequelae are permanent or reversible. The main factor contributing to this knowledge gap is that there is meager research on the late-developing and long-term neurological effects of acute H_2_S poisoning. Apparently, this knowledge gap also exists for acute cyanide poisoning, another highly toxic chemical that shares similar toxic mechanisms with H_2_S. There are few studies in which patient follow-up was performed 1–10 year after hospital discharge, which is essential to determine long-term neurological sequelae. In many cases, neurological sequelae were simply not assessed. Also, in cases where neurological and neuropsychological sequelae were assessed, the technique used varied, and the process was not standardized. With respect to animal models of H_2_S-induced brain injury, we observed that most of the animal models in the published literature failed to recapitulate the typical natural exposure scenario, i.e., via the inhalation route. Several investigators used the IP/IV injection of inorganic chemical donors of H_2_S, bypassing or excluding the contributions of the lung–brain and the nose–brain axes to brain injury and neurological sequelae. Therefore, research using appropriate inhalation animal models is desired. Considering that neurological diseases currently impart a heavy burden on patients, their providers, and the healthcare system, and since the population of individuals with these conditions is steadily increasing annually, it is critical that we understand the role of the environment in the pathogenesis of these conditions, including the role of acute cyanide and H_2_S poisoning, in order to develop targeted intervention strategies to reduce the burden of neurological diseases.

## 9. Future Perspectives

Recommendations for future research directions needed to move this field forward include long-term (at least 10 year) interdisciplinary evaluations and assessment of victims of acute H_2_S intoxication by qualified medical experts in radiology (imaging), neurology, neuropsychology, and neuropsychiatry to fully characterize the neurological sequelae of this potent gas in surviving victims. Research using animal models should also involve transdisciplinary collaborations, using animal models that recapitulate the inhalation route of exposure, and should be of sufficient duration to allow for delayed effects to develop and to determine whether these effects are reversible or not. It is also noteworthy that the published literature is devoid of molecular mechanistic research deciphering the molecular mechanisms of how neurological sequelae develop. In addition, the role of the lung–brain axis in the pathogenesis of H_2_S-induced neurological sequelae needs further research. The contribution of this route to the development of sequelae reported in this case should not be dismissed, especially considering that the lung is directly impacted by H_2_S gas via inhalation. It should be noted, however, that some neurological sequelae were reported, while anatomic imaging scans did not show any abnormalities. This suggests that some sequelae may not be triggered by the presence of brain lesions but rather functional or biochemical changes. It should also be noted that the sequelae induced by H_2_S are not identical to those caused by cyanide, azide, hypoglycemia, or asphyxia (hypoxia). This suggests that H_2_S poisoning bears unique toxic properties not shared by other toxicants, with some shared toxic mechanisms, e.g., inhibition of the CCO enzyme.

## Figures and Tables

**Table 1:** A summary of the literature reporting neurological sequelae in victims of acute H_2_S poisoning. N/A = not available.

Reference Title	Authors	Age	Exposure Duration	Comments
Air pollution by H_2_S in Poza Rica, Mexico; an evaluation of the incident of 24 November 1950	McCabe and Clayton 1952 [39]		Up to 20 min	Four survivors (age not given) developed neurological sequelae; two survivors developed neuritis of the acoustic nerve; one survivor developed dysarthria; and the fourth survivor developed aggravated epilepsy.
Poisoning by sewer gas with unusual sequelae	Hurwitz 1954 [31]	46	30 min	A 30 min exposure in a 46 year old male manifested as neurological sequelae 3 months after exposure, with exaggerated reflexes and tremors.
Hydrogen sulfide poisoning: a review of 5 years’ experience	Burnett WW, et al., 1977 [37]	19–31	N/A	A review of 221 cases. The average age was 31 year old. All cases involved workers, with 43% between 21 and 30 year old. A low prevalence of sequelae was reported, but no specialized neurological or neuropsychiatric examinations were performed.
Neurological sequelae of massive hydrogen sulfide inhalation	Matsuo F et al., 1979 [35]	45	N/A	A 45 year old male was rendered unconscious and entered a chronic vegetative state. A CT scan showed bilateral cerebral hemispheres and lentiform nucleus lesions. He died 5 weeks after exposure despite treatment.
Health implications of occupational exposures to hydrogen sulfide	Arnold IMF, et al., 1985 [38]	19–30	N/A	A 5 year retrospective study was conducted using Compensation Board records of 250 workers: 54.6% were 21–30 year old; 98% were male. Compensation was given to 58 cases (23.2%) due to lost time from work of <1 mo duration. Completeness of the recovery of individuals could not be determined. No specialized neurological or neuropsychiatric examinations were performed.
Hydrogen sulfide poisoning from toxic inhalations of roofing asphalt fumes	Hoidal CR, et al., 1986 [28]	35	20	A 35 year old male exposed for 20 min entered a chronic vegetative state despite 100% oxygen therapy.
A review of 152 cases of acute poisoning of hydrogen sulfide	Wang DX, 1989 [43]	18–57	Not given	A large population study of 120 males and 32 females, with an age range of 18–57 year old. A total of 95 patients were followed for 1–10 years; 39 of them (41%) showed neuropsychiatric sequelae.
Brain damage caused by hydrogen sulfide: A follow up study of six patients	Tvedt B. 1991 [44]	30–59	5–20 min	All males, 30–59 year old, who were rendered unconscious for 5–20 min showed persistent neurological impairment at neurological and neuropsychological re-examination 5–10 year after the accident. The authors stressed importance of long-term follow-up in order to identify neurological sequelae.
Delayed neuropsychiatric sequelae after acute H_2_S poisoning: affection of motor, memory, vision and hearing	Tvedt B, et al., 1991 [26]	31	15–20 min	The 5-year follow-up of a 31 year old male exposed for 15–20 min showed cerebral atrophy and widening of the lateral ventricle (MRI and CT). Motor, memory, vision, and hearing impairment were also noted. The authors concluded that sequelae may be more common than previously reported
Case report: Profound neurobehavioral deficits in an oil field worker overcome by hydrogen sulfide	Kilburn KH, 1993 [34]	24	10 min	A 24 year old male rendered unconscious after a 10 min exposure was treated with oxygen and released 30 min later. The subject manifested profound cognitive, memory, and neuropsychological deficits 49 months postexposure.
Acute poisoning caused by hydrogen sulphide: clinical features of 3 cases	Sanz-Gallen et al., 1994 [30]	23–28	50–60 min	Of three 23–28 year old males exposed for 50–60 min, one entered a vegetative state, and the second developed neurologic sequelae.
Occupational Fatality and persistent neurological sequelae after mass exposure to hydrogen sulfide	Snyder JW et al., 1995 [45]	24–50	Seconds to unknown time	A case report of acute H_2_S poisoning, including 37 people affected (age range 24–50 year), 6 admitted, and 1 death. At least one patient who underwent hyperbaric oxygen treatment developed neurological sequelae. The authors recommended that victims of acute H_2_S poisoning presenting coma or evidence of neurotoxicity should undergo baseline and annual neurological and neuropsychological testing for at least 5 year because patients with long-term neurological sequelae continue to be reported. This is necessary in order to detect permanent alterations in the nervous system following acute H_2_S exposure.
Persistent cognitive and motor deficits following acute hydrogen sulfide poisoning	Schneider JS et al., 1998 [36]	27	N/A	A 27 year male was unconscious and treated with hyperbaric oxygen for several days. At 3 year after the accident, PET showed abnormal metabolism in basal ganglia, thalamus, and temporal and inferior parietal lobes. Neurobehavioral, neuropsychological, and neurofunctional impairment.
Cognitive sequelae three months after hydrogen sulfide poisoning	Fenga C et al., 2002 [33]	36	A few mins	A 36 year old male was exposed to 500 ppm for a few minutes. Three months postexposure, he manifested reduced cognition, depression, and personality changes even though a neurological exam and neuroimaging were unremarkable.
Hydrogen sulfide exposure without loss of consciousness: chronic effects in four cases	Hirsch AR 2002 [29]	N/A	2.5 h	Four workers (sex and age not stated) had persistent neuropsychiatric disorders and abnormal P300 evoked responses 1 year after exposure.
Neurological sequela of hydrogen sulfide poisoning	Nam B et al., 2004 [27]	25	10 min	A 25 year old male exposed for 10 min was hospitalized in a coma. Necrosis of basal ganglia and motor cortex were identified by MRI 30 days postexposure. Neurological cognitive deficits were identified up to 5 mo postexposure.
Acute hydrogen sulfide poisoning in a dairy farmer	Gerasimon G et al., 2007 [40]	36	5 min	A 36 year old male exposed for 5 min showed MRI lesions in superior the cerebral hemispheres, basal ganglia, and thalamus. Problems with balance, dysarthria, and difficulty eating for several months improved with intense neuro-rehabilitation.
Hydrogen sulfide inhalation toxicity at a petroleum refinery in Sri Lanka	Shivanthan M, et al., 2013 [32]	36	10 min	Dysarthria, status epilepticus and retrograde amnesia were identified in one 36 year old male survivor after a 10 min exposure.
Analysis of CT and MR imaging features of the brain in patients with hydrogen sulfide poisoning based on clinical symptom grading	Tang D et al., 2022 [46]	18–54	N/A	A retrospective analysis of CT and MRI data from 40 patients (35 males, 5 females, age 18–54 year, median age 37.5 year) with acute poisoning clinically graded according to central nervous system score (minor *n* = 10, moderate *n* = 17, severe *n* = 13). Generalized brain edema was found in most severe cases and in one moderate case. Symmetrical abnormal intensities in basal ganglia and around lateral ventricles were seen in most of the severe cases. Subarachnoid or intracerebral hemorrhage and cerebral tonsillar herniation were present is a few severe cases that also had a poor prognosis. Brain lesions were still present in some patients 5.5 months after exposure. One victim who was exposed for 50 min developed severe neurological sequelae.

**Table 2:** A summary of the literature reporting neurological sequelae in victims of acute cyanide poisoning.

Reference Title	Authors	Age	Exposure Dose	Comments
Clinical and CT scan findings in a case of cyanide intoxication	Grandas 1983 [47]	39	Unknown	A 39-year-old man developed brain lesions in basal ganglia and the front cortex 1 month after exposure and putamen and external globus pallidus 2 to 3 years after exposure. The patient manifested delayed neurological sequelae, including signs of Parkinsonism (bradykinesia, resting tremor, and postural instability). His neurological sequelae were permanent.
Extrapyramidal disturbances after cyanide poisoning (first MRT investigation of the brain)	Messing 1991 [48]	29	500 mg of potassium cyanide	A 29-year-old man developed delayed dysarthria and signs of Parkinsonism, including bradykinesia and monosynaptic reflexes. CT scans and MRI imaging revealed lesions in the basal ganglia 5 months after attempting suicide.
Chronological changes of MRI findings on striatal damage after acute cyanide intoxication: pathogenesis of the damage and its selectivity, and prevention for neurological sequelae: a case report	Kasamo 1993 [49]	31	20–40 g of potassium cyanide	A patient developed delayed neurological sequelae characterized by lesions restricted to the caudate nuclei and putamina using MRI. The lesions in putamina were still present at 9 months after exposure to cyanide.
Neurological sequelae of cyanide intoxication—the patterns of clinical, magnetic resonance imaging, and positron emission tomography findings	Rosenow 1995 [50]	22 and 43	1.3 g of potassium cyanide for a 22-year-old man Unknown amount for a 43-year-old man	Both patients had delayed extrapyramidal motor and cerebral symptoms, including bradykinesia, months after exposure to cyanide. MRI imaging showed lesions in the globus pallidus, putamen, substantia nigra, subthalamic nucleus, and cerebellum. A 22-year-old patient showed reduced glucose metabolism and dopamine uptake in the putamen and caudate. The 22-year-old patient recovered 3 years after exposure to cyanide, whereas the 43-year-old man still had brain lesions in the pallidum, posterior putamen, and substantia nigra 5 years after exposure to cyanide.
Accidental choke-cherry poisoning: early symptoms and neurological sequelae of an unusual case of cyanide intoxication	Pentore 1996 [51]	56	Unknown	A 56 year old woman experienced breathing difficulty and entered coma. For 2 weeks, she had neurological symptoms including confusion, disorientation, and agitation. About 30 days post-admission, she developed signs of Parkinsonism and reduced bilateral visual acuity. The patient fully recovered at 15 months after admission.
MR changes after acute cyanide intoxication	Rachinger 2002 [52]	35	Unknown	A 35 year old female immediately went into a coma after cyanide poisoning and showed agitation and akinetic mutism after withdrawing sedation and removal of respiratory support in hospital. Six weeks after cyanide poisoning, MRI imaging revealed hemorrhage and hyperintense signal change in the striatum, globus pallidus, and basal ganglia.
Surviving acute cyanide poisoning: a longitudinal neuropsychological investigation with interval MRI	Mohan 2014 [53]	22	Unknown	A 22-year-old woman developed neuropsychological effects after ingestion of cyanide. Around day 5 post-ingestion, she had deficits in episodic memory. She was assessed to have impaired episodic memory and retrograde amnesia. She also had attention deficits. The patient did not have movement disorder. MRI neuroimaging revealed deficits in the basal ganglia, thalamus, and cerebral hemispheres and edema in the hippocampus. At 5 months, these lesions were still present, albeit smaller, but the hippocampus was reported to have atrophied. A neuropsychological assessment at 3 weeks post-injury revealed reduced cognitive functioning and problem-solving skills. These defects were significantly reversed at 5 months.
Long-term neuropsychiatric sequelae in a survivor of cyanide toxicity patient with arterialization	Alqahtani 2020 [54]	45	Unknown inhalation	A 45-year-old woman developed cognitive deficits, dysarthria, dystonia, and altered sleep patterns 3 months after cyanide poisoning. The authors emphasized conducting full neurological and intellectual evaluations to find neuropsychiatric sequelae in cases of acute cyanide poisoning. In this case, the CT scan and MRI were normal.

**Table 3:** A summary of the potential drugs for treating acute H_2_S poisoning.

Drug	Mode of Action
Methemoglobin inducers	Sodium nitrite induces the formation of methemoglobin heme in vivo, which reduces the inhibition of cytochrome c oxidase [93]. It was also proposed that methemoglobin inducers may serve as a NO donor, which reverses the sulfide inhibition of cytochrome c oxidase [72].
Epinephrine	Epinephrine with chest compressions counteracts sulfide-induced pulseless electrical activity (PEA) [67].
Hydroxocobalamin	Hydroxocobalamin binds to sulfide in the blood to prevent free sulfide from entering tissues [94].
Cobinamide	Cobinamide is an analog of hydroxocobalamin and a precursor in hydroxocobalamin synthesis. Cobinamide has more sulfide binding sites than hydroxocobolamine [95].
Midazolam	Midazolam is an anticonvulsant drug and a potential antidote to counteract sulfide-induced epileptic activity. The prevention of seizure-like activity prevents mortality and reduces neuropathology in mice [18].
Methylene blue	Methylene blue is potential antioxidant drug. It has shown efficacy in the treatment of methemoglobinemia. Methylene blue is reported to restore cellular redox potential, mitochondrial function, and cardiac myocyte function [71,96]. It has also shown efficacy in reducing H_2_S-induced neurological sequelae in a rat model.
Hyperbaric oxygen	Hyperbaric oxygen treatment provides supplemental oxygen to reduce the hypoxic effects of sulfide [97].

## Data Availability

Not applicable.

## Abbreviations

The following abbreviations are used in this manuscript:

H_2_S	hydrogen sulfide
ATP	adenosine triphosphate
CCO	cytochrome c oxidase
FDA	Food and Drug Administration
MRI	magnetic resonance imaging
PET	positron emission tomography
CT	computed tomography
SPECT	single-photon emission computed tomography
ALI	acute lung injury
